# Transcriptomic phases of periodontitis lesions using the nonhuman primate model

**DOI:** 10.1038/s41598-021-88803-6

**Published:** 2021-04-29

**Authors:** Jeffrey L. Ebersole, Radhakrishnan Nagarajan, Sreenatha Kirakodu, Octavio A. Gonzalez

**Affiliations:** 1 Department of Biomedical Sciences, School of Dental Medicine, B221, University of Nevada Las Vegas, 1001 Shadow Lane, Las Vegas, NV 89106 USA; 2 Center for Oral and Systemic Disease, Marshfield Research Foundation, Marshfield, WI USA; 3 Center for Oral Health Research College of Dentistry, University of Kentucky, Lexington, KY USA; 4 Division of Periodontology, University of Kentucky, Lexington, KY USA

**Keywords:** Microbiology, Bacteria, Bacterial pathogenesis, Inflammation, Immunology, Infectious diseases

## Abstract

We used a nonhuman primate model of ligature-induced periodontitis to identify patterns of gingival transcriptomic after changes demarcating phases of periodontitis lesions (initiation, progression, resolution). A total of 18 adult *Macaca mulatta* (12–22 years) had ligatures placed (premolar, 1st molar teeth) in all 4 quadrants. Gingival tissue samples were obtained (baseline, 2 weeks, 1 and 3 months during periodontitis and at 5 months resolution). Gene expression was analyzed by microarray [Rhesus Gene 1.0 ST Array (Affymetrix)]. Compared to baseline, a large array of genes were significantly altered at initiation (n = 6049), early progression (n = 4893), and late progression (n = 5078) of disease, with the preponderance being up-regulated. Additionally, 1918 genes were altered in expression with disease resolution, skewed towards down-regulation. Assessment of the genes demonstrated specific profiles of epithelial, bone/connective tissue, apoptosis/autophagy, metabolism, regulatory, immune, and inflammatory responses that were related to health, stages of disease, and tissues with resolved lesions. Unique transcriptomic profiles occured during the kinetics of the periodontitis lesion exacerbation and remission. We delineated phase specific gene expression profiles of the disease lesion. Detection of these gene products in gingival crevicular fluid samples from human disease may contribute to a better understanding of the biological dynamics of the disease to improve patient management.

## Introduction

Periodontal diseases are chronic dysregulated responses to dysbiotic microbiomes^1–3^. However, the literature is sparse regarding the earliest changes that occur in the local microbiome at sites that will transition from health to periodontal lesions. Furthermore, the disease is expressed temporally as exacerbations and remissions with the frequency and magnitude of the episodes explaining the variation in disease extent/severity across the population^4,5^. This episodic disease can occur at one or multiple sites sequentially or concomitantly, and appears to occur with greater incidence at sites that have already demonstrated a previous disease process^6–8^.

A challenge in the field is that sampling of humans for the comprehensive biology of the disease process remains somewhat problematic for detailing the dynamics of an episodic disease. The experimental designs are generally limited to a point-in-time with clinical definition of health or disease at that point. While human disease can be clinically monitored over time at multiple sites per tooth and across the dentition, the inability to sample site-specific tissues on multiple occasions, makes the assessment of the biology of the tissues representing differences in phases of initiation, progression, stabilization, or resolution of the disease lesion at a particular site challenging. Additionally, while investigations of periodontitis in rodents and rabbits^9,10^ clearly can provide useful information regarding the underlying biology of local and systemic host responses, the autochthonous complex microbiomes are completely different in these species versus humans, and the major oral pathogens in human disease have no tropism for colonizing rodents and rabbits^11–13^. Thus, we and others have employed a nonhuman primate model of periodontal disease over the last nearly 40 years. It has been shown that the microbiology, immunology, and clinical expression of periodontitis are quite similar in humans and nonhuman primates, including naturally-occurring disease that increases with aging^14–16^. We have also recently reported a familial relationship of periodontitis susceptibility and resistance in multigenerational matrilines of *Macaca mulatta*^14^ consistent with some heritability/genetic contribution to the disease, as has been described in humans^17–19^.

Thus, this report describes longitudinal studies of biological processes of periodontitis using a nonhuman primate model of ligature-induced disease. Gene expression profiles were determined in gingival tissues with health and during initiation, progression, and resolution of periodontitis, reflecting the episodic nature of the disease in humans. The hypothesis tested was that there would be unique gene expression profiles that discriminate phases of periodontitis. The findings would shed some light into the temporal nature of changes in biological factors/pathways through the disease process. This would provide the potential for identifying targeted biomolecules that could be used to better characterize disease sites in humans, and identify differences in the biological characteristics of healthy (never diseased) sites from previously diseased sites that have been successfully treated, as potential biomarkers of future risk for disease.

## Results

### Dynamics of periodontitis lesions

**Figure 1:**
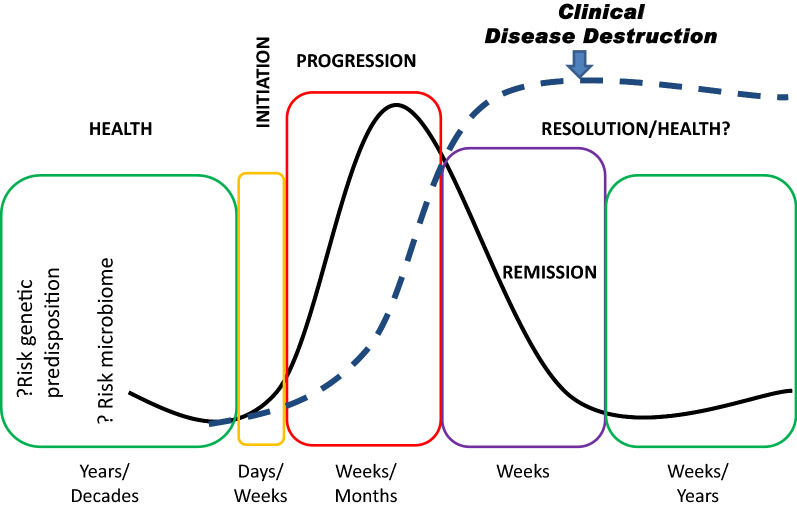
Model of exacerbations and remission of periodontal disease lesions. Graph reflects inherent risk due to genetic predisposition and existing oral microbiome characteristics. Biologic changes occur with initiation and progression of disease the generally presage the clinical changes measured. The model indicates that the biological parameters of disease stabilize and decrease during disease remission and resolution. However, the clinical features of lost epithelial attachment, connective tissue destruction and alveolar bone resorption remain as markers of the previous disease process.

Figure 1 provides a schematic model of periodontitis as suggested from human studies of exacerbation and remission of clinical features of disease. It is well recognized that periodontitis is generally not expressed until about the 3rd–4th decade of life, even though the host response system and oral microbiome are interacting during a 30 + year time period prior to disease^20–22^. Existing data support that many or most of the bacteria generally associated with periodontitis are also present in younger individuals, who often demonstrate substantial gingival inflammation, but do not appear to transition to destructive disease^23–27^. Additionally, numerous reports have suggested a genetic contribution to disease related to gene polymorphisms that would exist from the beginning of life in at-risk individuals^17^. Thus, the “risk microbiome’ and “risk genetic predisposition” occur for decades prior to clinical disease expression. At some point within an individual at one or more sites a disease process is initiated. Based upon the biology of how a host reacts to a bacterial challenge, this initial insult would likely last for days or weeks, either resolving rapidly or transiting to progressing disease. It is unknown regarding the temporal linkage of the biological changes with detectable clinical disease, but it would be predicted that this interaction occurs with disease progression over weeks to months based upon rodent and nonhuman primate experimental data^9,10,28^. While once clinical tissue destruction has occurred, it is irreversible, human studies would support that the disease can and does resolve biologically, limiting the extent of destruction^4,29,30^. With a particular disease lesion, this now stabilized site may exist for weeks or even years; however, human studies support that the greatest predictor of formation of a periodontal lesion is past disease at the same site^5,31^. Nevertheless, there is minimal guidance regarding the biology of resolved sites relative to future disease exacerbations. Thus, we implemented a study to examine this temporal nature of the disease process in nonhuman primates, focusing on gingival gene expression profiles to identify unique biologic processes occurring at the different stages of disease.

**Figure 2:**
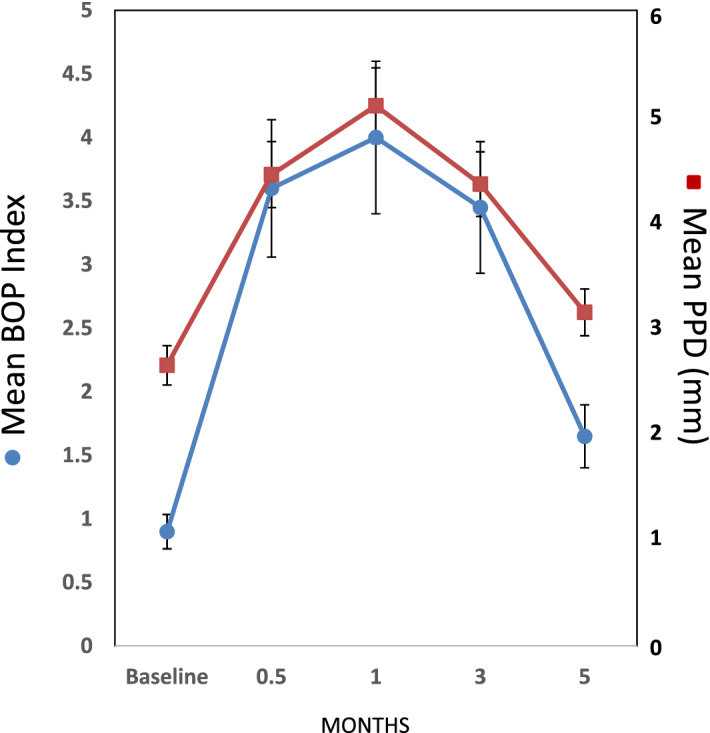
Clinical features of inflammation (bleeding on probing, BOP) and destructive disease (probing pocket depth, PPD) in ligated sites of the nonhuman primates. Ligatures were removed after clinical measures at 3 months, with 5 month samples representing clinical resolution. The points denote the mean values from 18 animals and the vertical brackets enclose 1 SD.

Figure 2 summarizes the clinical presentation of ligated teeth for bleeding on probing and probing pocket depth. Consistent with previous studies, inflammation and tissue destructive changes occurred rather rapidly following initiation of the challenge (i.e. 0.5 months), and continued to progress over 1–3 months. Removal of the ligatures after sampling at 3 months resulted in a general clinical resolution of these disease features that approximated the baseline health values.

### Gene expression profiles in periodontitis lesions

A detailed examination (Table 1) showed a substantial number of genes with significant differences from baseline samples. These included genes that were both up- and down-regulated at ≥ 1.5-fold with disease initiation and progression. Interestingly, while there was a large number of genes that were significantly different between disease resolution and baseline, only about 400 demonstrated this fold-level of differential expression.

**Table 1:** Distribution of altered gene expression in gingival tissues during ligature-induced periodontitis and at disease resolution compared to baseline healthy tissues.

Sample (mo.)	Stage	P-value < 0.01	Gene expression changes (#)	
		Total#	UP (Fold ≥ 1.5)	DOWN (Fold ≥ 1.5)
0.5	Initiation	6049	943	472
1	Early progression	4893	692	471
3	Late progression	5078	542	372
5	Resolution	1918	176	221

**Figure 3:**
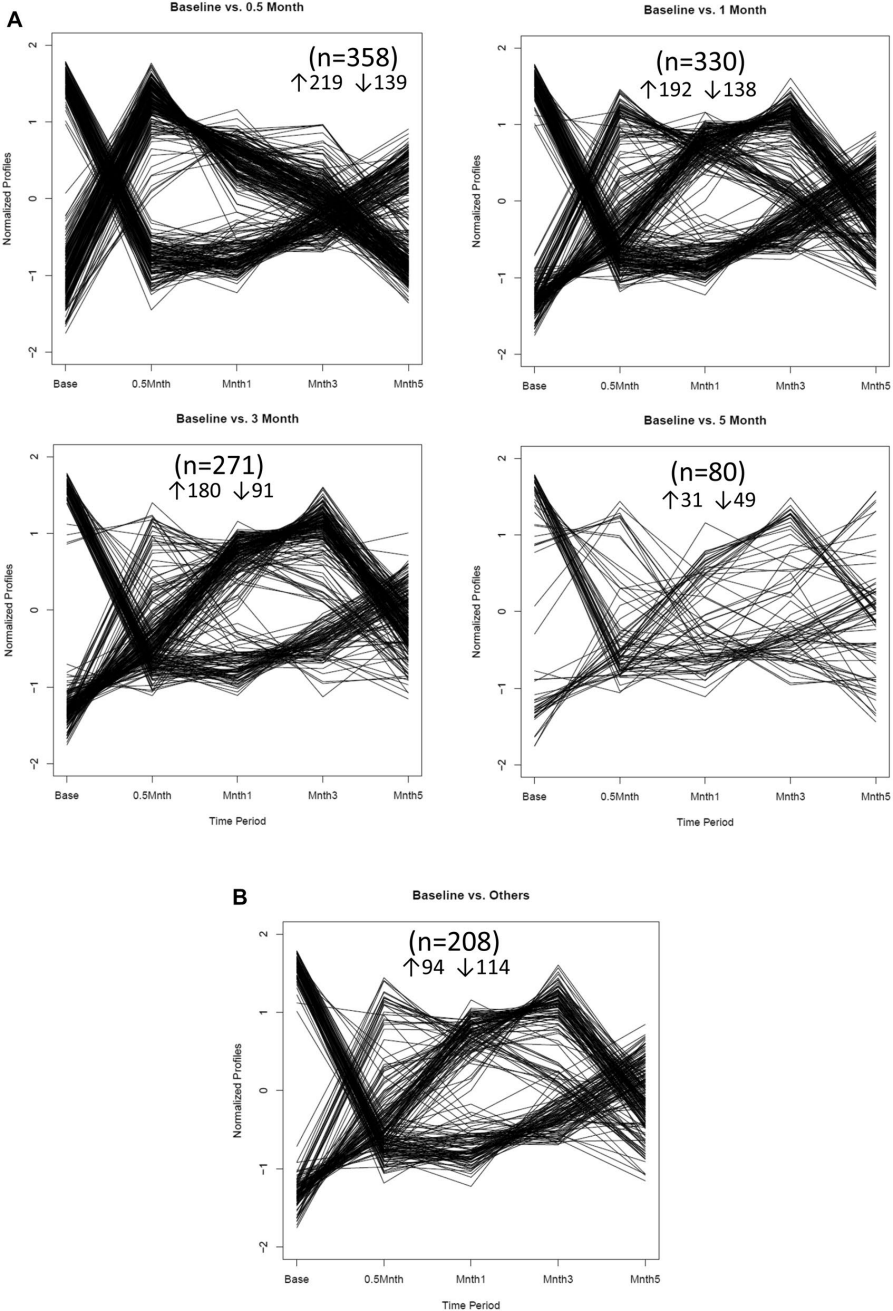

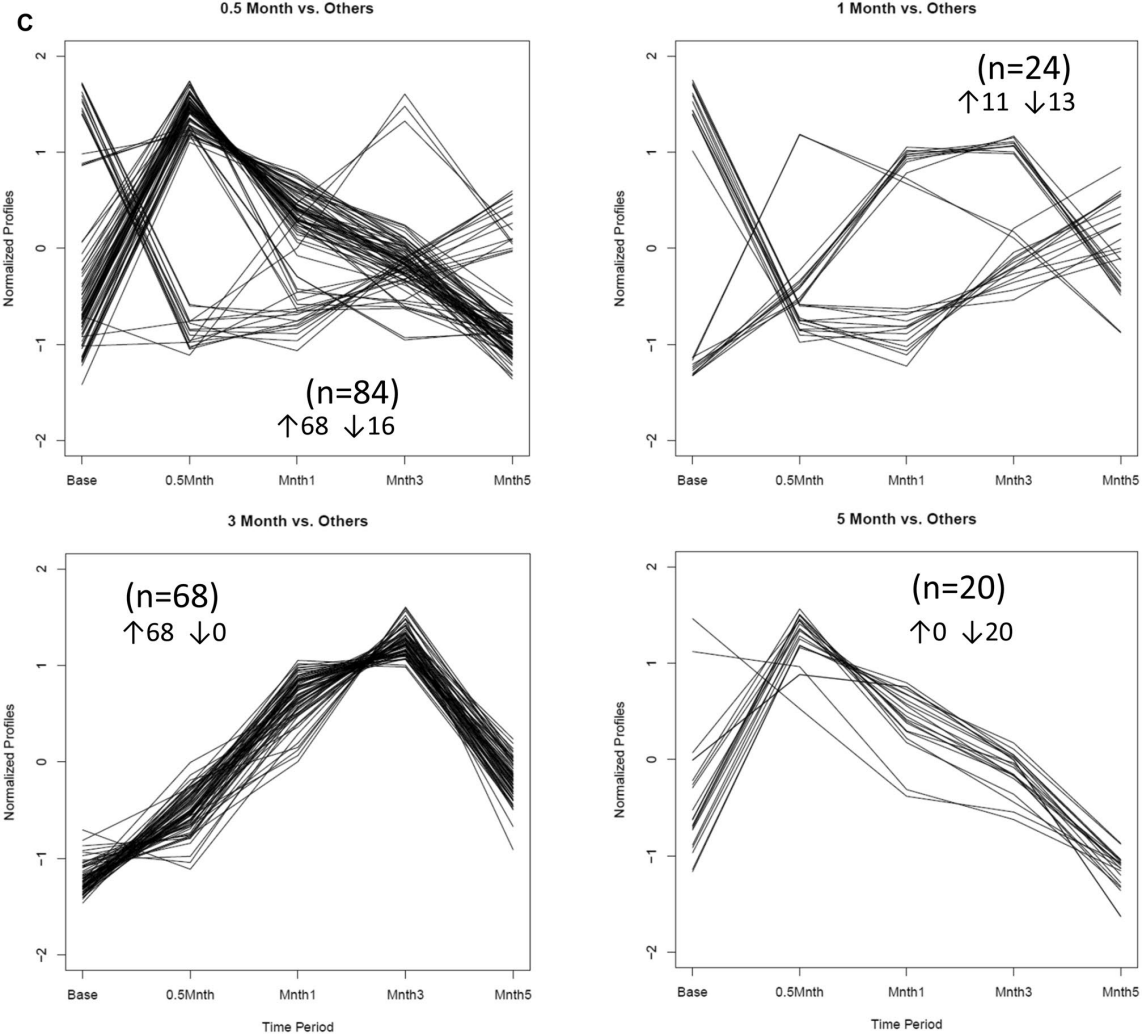
(**A**) Patterns of gene expression with disease and resolution compared to baseline/health. (**B**) Patterns of gene expression in baseline/health versus all other time points. (**C**) Patterns of gene expression with disease and resolution compared all other time points in the model. Numbers in parentheses denote number of genes within the particular patterns, and whether the patterns denoted up (↑) or down-regulation (↓) of the gene experssion.

Since a goal was to identify genes that were uniquely expressed at each time point, the plots in Fig. 3 provide the features of expression for subsets of the genes for baseline expression compared to the other time points. As shown, 358, 330, 271, and 80 genes at 0.5, 1, 3 and 5 months, respectively that fulfilled the threshold of ≥ 1.5 difference between baseline and any other time point. Of interest was that during disease these genes were up-regulated, while at resolution the distinctive genes were expressed at a lower-level than baseline. Figure 3c depicts a similar analysis of gene expression during disease initiation, progression and resolution compared to all other phases. This differential expression showed a limited number of genes (n = 20) and (n = 24) that were distinct at 0.5 and 1 month, respectively. At 3 months (late progression), 68 genes showed increased or decreased expression by > twofold. As noted with the baseline comparison the primary effects on gene expression with resolution was a profile of significantly (*p* < 0.05) decreased expression of genes (n = 18) compared to the other time points.

### Functional characteristics of gene expression of periodontitis lesions

**Figure 4:**
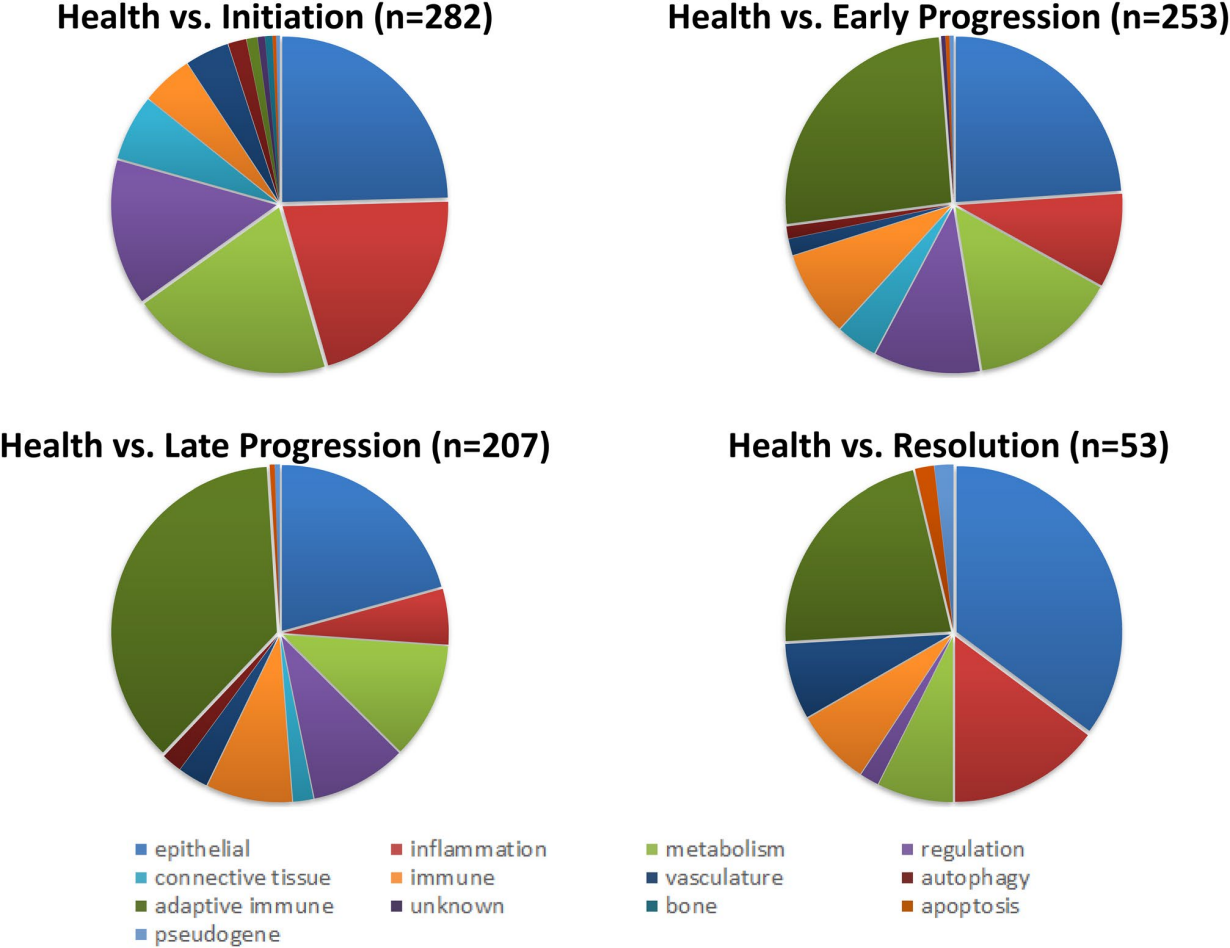
Depiction of the array of genes in various functional categories that differed by ≥ twofold at health versus other sampling points. Pie charts denote the proportions of each functional category of genes that comprised the overall number of genes (in parentheses) that were differentially expressed.

The next step in the process was to identify those genes/pathways that provided unique signatures for baseline healthy tissues, tissues from sites with initiation and progression of disease, and tissue samples from clinically resolved lesions. Figure 4 summarizes the functional categories of the genes that were increased or decreased by > twofold at each of the phases compared to baseline healthy tissues. Numerous genes related to epithelial cell biology were affected as early as 2 weeks (Initiation) and remained different from health throughout lesion progression and even in resolution tissues. Inflammation genes were overly represented at disease initiation; decreasing in number with disease progression. Additionally, cellular metabolic and regulatory genes were affected rapidly at disease initiation and early progression and decreased in representation in late progression and resolution. Finally, adaptive immune genes were highly represented in early and late disease progression, with many remaining altered even in resolution samples. Table 2 provides a summary of the gene expression profile functions that were phase-related across health, disease and resolution samples. In healthy tissues, epithelial cell, metabolic, and regulatory genes generally were elevated and down-regulated with disease. In contrast, genes related to adaptive immune responses were expressed at low levels in healthy tissues and increased significantly in prevalence with disease. With disease initiation, the number of inflammatory genes were substantially increased as were changes in additional genes related to epithelial cell functions and integrity of the epithelium. Early progression showed a more limited number of genes that were differentially expressed (> twofold) compared to all other time points, primarily for epithelial cell genes (decreased) and adaptive immune genes (increased). A distinctive gene profile was observed during late progression, with 96% of the phase specific genes associated with adaptive immune responses. Finally, in resolution samples, a low number of unique differentially expressed genes was observed with expression of primarily inflammation-associated genes remaining elevated.

**Table 2:** Identification of altered gene expression in gingival tissues, with functional patterns unique to each phase of health, disease and resolution by comparison to gene expression profiles in all other phases of lesion formation and resolution.

Phase	Total	Functional categorization											
		Epi	Meta	Regul	Inflam	Imm	Adap	Conn	Vasc	Auto	Apop	Bone	Pseudo
Health/BL	163	45	21	13	10	13	49	6	2	2	0	0	1
Initiation/2 wks	70	13	7	8	23	1	3	7	5	0	0	0	0
Early progression/1 mo	20	10	0	1	1	0	7	1	0	0	0	0	0
Late progression/3 mo	50	0	0	1	1	0	48	0	0	0	0	0	0
Resolution/5 mo	17	1	1	2	10	0	0	2	1	0	0	0	0

Value denote number of genes represented in each category.

Fxn denotes functional categorization of genes: *Epi* epithelium, *Conn* connective tissue, Bone, *Meta* metabolic, *Regul* regulatory, Inflam –inflammation, *Imm* innate immune, *Adap* adaptive immune, *Auto* autophagy, *Apop* apoptosis, Pseudo—pseudogene.

A listing of genes that specifically hallmarked healthy gingival tissues from the stages of disease or resolved lesions is provided in Table 3. Of these 43 genes, those related to epithelial cells, were generally expressed in significantly elevated levels in healthy tissues and decreased with onset of disease. In contrast, cellular metabolism, regulatory, and inflammation/immune genes were increased with disease onset and progression. Table 3 also provides a similar summary identification of 45 genes that hallmarked disease initiation/early progression of the lesion. These genes were generally up-regulated during these stages of disease and were represented by a broad mix of functions, generally significantly increased over baseline healthy levels. Examination of unique phase specific gene expression profiles during late progression of disease identified 45 altered transcript signals (Table 3). These were skewed towards genes related in inflammation and immune responses. As with gene expression at initiation/early progression, these identified genes were increased compared to baseline samples. However, the most prominent markers comprising > 50% of the up-regulated genes were associated with immunoglobulin formation and antibody recombination processes of adaptive immune responses. Finally, we identified gene differences (n = 33) in baseline healthy sites versus sites that appeared clinically healthy post-resolution of a disease process (Table 3). Of note was that a number of these genes were decreased from health with disease and continued at a lower level of expression in the resolution samples, albeit, at levels improved over the disease samples (e.g. keratins, LIPM). MUC4 was unique in that it was increased with disease, but increased to even greater levels once the disease had resolved. In contrast, EGR1 was decreased with disease from health and was down-regulated even more in resolution samples. Additionally, some genes, e.g. CD36, PTGS2 genes were decreased only in resolved disease tissues versus health or disease. No differences were noted in these gene expression profiles based on sex of the animals. While not included in the table, an observation was that of the array of Ig genes of adaptive immunity that were significantly elevated in late progression, remained elevated in resolution compared to healthy tissue samples.

**Table 3:** Identification of altered gene expression in gingival tissues: comparing healthy versus diseased gingival tissues (n = 43; in all cases levels of gene expression were elevated in healthy tissues); during initiation/early progression of disease (n = 45); during late progression of disease (n = 46); and comparing baseline healthy tissues to clinically resolved lesions (n = 33).

Gene ID	FXN	Ontology
**Health versus disease**		
ANAX3	Conn	Annexin A3
DRAM1	Conn	DNA damage regulated autophagy modulator 1
MMP1	Conn	Matrix metalloproteinase 1
PXDN	Conn	Peroxidasin
SERPINE2	Conn	Serpin family E member 2
TFPI	Conn	Tissue factor pathway inhibitor
ALOXE3	Epi	Arachidonate lipoxygenase 3
ALOX12B	Epi	Arachidonate 12-lipoxygenase, 12R type
CDSN	Epi	Corneodesmosin
CRNN	Epi	Cornulin
DSC1	Epi	Desmocolin 1
KLK5	Epi	Kallikrein related peptidase 5
KRT1	Epi	Keratin 1
KRT2B	Epi	Keratin, type II cytoskeletal 2 oral-like
LCE3C	Epi	Late cornified envelope 3C
NID1	Epi	Nidogen 1
ODAM	Epi	Odontogenic, ameloblast associated
PRR9	Epi	Proline rich 9
RPTN	Epi	Repetin
SERPINB13	Epi	Serpin family B member 13
SPINK9	Epi	Serine peptidase Inhibitor, Kazal Type 9
BPIL2/BPILFC	Imm	Bactericidal/permeability increasing protein-like 2
SIRPB1	Imm	Signal regulatory proten beta 1
SLAMF6	Imm	SLAM family member 6
WFDC12	Imm	WAP four-disulfide core domain 12
CD177	Inflam	CD177 molecule
CSF3R	Inflam	Colony stimulating factor 3 receptor (granulocyte)
CXCL8	Inflam	Interleukin 8
CXCL6	Inflam	Chemokine (C-X-C motif) ligand 6
GSDMA	Inflam	Gasdermin A
HPGD	Inflam	15-Hydroxyprostaglandin dehydrogenase
IL36B/IL1F8	Inflam	Interleukin 36 beta
SERPINB5	Inflam	Serpin family B member 5
TNFRSF19	Inflam	TNF receptor superfamily member 19
AADAC	Metab	Arylacetamide deacetylase
AKR1C3	Metab	Aldo–keto reductase family 1 member C3
ALAS2	Metab	5′-aminolevulinate synthase 2
ARSF	Metab	Arylsulfatase F
ESYT3	Metab	Extended Synaptotagmin 3
SERPINA3	Metab	Serpin family A member 3
TDH	Metab	L-threonine dehydrogenase (pseudogene)
FAM178B	Regul	Family with sequence similarity 178 member B
RNU6-1	Regul	U6 spliceosome
**Initiation/early progression**		
BLK	Adap	BLK proto-oncogene, Src family tyrosine kinase
IGKV1-ACY*02	Adap	Immunoglobulin kappa variable 1 ACY*02
IGKV1S14*01	Adap	Immunoglobulin kappa variable 1S14*01
IGKV3-ACF*02	Adap	Immunoglobulin kappa variable 3 ACF*02
IGLV2S9*01	Adap	Immunoglobulin lambda variable 2S-9*01
MZB1	Adap	Marginal zone B and B1 cell specific protein
MUC4	Auto	Mucin 4, cell surface associated
NAIP	Auto	NLR family apoptosis inhibitory protein
RUBCNL	Auto	Rubicon like autophagy enhancer
ACTA2	Epi	Actin alpha 2, smooth muscle
ADAM12	Epi	ADAM metallopeptidase domain 12
KRT8	Epi	Keratin 8
KRT15	Epi	Keratin 15
NID2	Epi	Nidogen 2 (osteonidogen)
PLIN2/ADFP	Epi	Perilipin 2
COL4A2	Conn	Collagen type IV alpha 2
ESM1	Conn	Endothelial cell-specific molecule 1
HAS2	Conn	Hyaluronan synthase 2
PLAU	Conn	Plasminogen activator, urokinase
PLAT	Conn	Plasminogen activator, tissue type
ADAMTS9	Metab	ADAM metallopeptidase with thrombospondin type 1 motif 9
CTSL	Metab	Cathepsin L
CYP4F3	Metab	Cytochrome P450 family 4 subfamily F member 3
NOX4	Metab	NADPH oxidase 4
MTHFS	Metab	Methenyltetrahydrofolate synthetase
MSMO1	Metab	Methylsterol monooxygenase 1
SLC11A1	Metab	Solute carrier family 11 member 1
SULF1	Metab	Sulfatase 1
TGM2	Metab	Transglutaminase 2 (C polypeptide, protein-glutamine-gamma-glutamyltransferase)
IL1B	Inflam	Interleukin 1 beta
MIR223	Inflam	microrna 223
NLRP12	Inflam	NLR family pyrin domain containing 12
SELL	Inflam	Selectin L
TLR2	Imm	TOLL like receptor 2
TLR4	Imm	Toll-like receptor 4
IL33	Imm	Interleukin 33
LILRA2	Imm	Leukocyte immunoglobulin like receptor A2
PLAC8	Imm	Placenta associated 8
CCDC9	Regul	Coiled-coil domain containing 9
GLI3	Regul	GLI family zinc finger 3
HGF	Regul	Hepatocyte growth factor
RGS18	Regul	Regulator of G protein signaling 18
SFRP4	Regul	Secreted frizzled related protein 4
OR6K6	Sig	Olfactory receptor family 6 subfamily K member 6
RTP3	Sig	Receptor transporter protein 3
VWF	UA	Von Willebrand factor
**Late progression**		
IGHG3*02	Adap	Immunoglobulin heavy constant gamma 3
IGHM	Adap	Immunoglobulin heavy constant mu
IGHV3-49	Adap	Immunoglobulin heavy variable 3–49
IGHV3-72	Adap	Immunoglobulin heavy variable 3–72
IGHV3-73	Adap	Immunoglobulin heavy variable 3–73
IGHV3-AGQ*02	Adap	Immunoglobulin heavy variable 3 AGQ*02
IGJ	Adap	joining chain of multimeric IgA and IgM
IGKV1-9	Adap	immunoglobulin kappa variable 1–9
IGKV1-ABV*02	Adap	Immunoglobulin kappa variable 1 ABV*02
IGKV1-ACY*02	Adap	Immunoglobulin kappa variable 1 ACY*02
IGKV1z	Adap	Immunoglobulin kappa variable 1z
IGKV2-ABW*04	Adap	Immunoglobulin kappa variable 2 ABW*04
IGKV2S19*01	Adap	Immunoglobulin kappa variable 2S19*01
IGKV3-ADU*02	Adap	Immunoglobulin kappa variable 3 ADU*02
IGKV4-1	Adap	Immunoglobulin kappa variable 4–1
IGLV1-ABB*02	Adap	Immunoglobulin lambda variable 1 ABB*02
IGLV1-ACV*02	Adap	Immunoglobulin lambda variable 1 ACV*02
IGLV2a	Adap	Immunoglobulin lambda variable 2a
IGLV2-ABU*02	Adap	Immunoglobulin lambda variable 2 ABU*02
IGLV3-AAV*04	Adap	Immunoglobulin lambda variable 3 AAV*04
IGLV5-AAX*02	Adap	Immunoglobulin lambda variable 5 AAX*02
IGLV7-46	Adap	Immunoglobulin lambda variable 7–46
IGLV8-61	Adap	Immunoglobulin lambda variable 8–61
KLHL6	Adap	Kelch like family member 6
COL4A2	Conn	Collagen type IV alpha 2
KRT24	Epi	Keratin, type I cytoskeletal 24-like
THBS1	Epi	Thrombospondin 1
BANK1	Imm	B cell scaffold protein with ankyrin repeats 1
FPR3	Imm	Formyl peptide receptor 3
GREM1	Imm	Gremlin 1, DAN gamily BMP antagonist
LST1	Imm	Leukocyte specific transcript 1
SERPINB5	Imm	Serpin family B member 5
TEK	Imm	TEK receptor tyrosine kinase
CD36	Inflam	CD36 molecule (thrombospondin receptor)
CXCR1	Inflam	Chemokine (C-X-C motif) receptor 1
PTGS2	Inflam	Prostaglandin-endoperoxide synthase 2
TREM1	Inflam	Triggering receptor expressed on myeloid cells 1
BCAT1	Metab	Branched chain amino acid transaminase 1
DYSF	Metab	Dysferlin
KYNU	Metab	Kynureninase-like
SLC2A14	Metab	Solute carrier family 2 member 14
SLC15A1	Metab	Solute carrier family 15 member 2
SNORD116-17	Regul	Small nucleolar RNA, C/D box 116–17
TENT5C	Regul	Terminal nucelotidyltransferase 5C
ZNF337	Regul	Zinc finger protein 337
UTS2B	Sig	Urotensin 2B
**Health versus resolution**		
IGLV2S9*01	Adap	Immunoglobulin lambda variable 2S-9*01
IGHV3-ADR*02	Adap	Immunoglobulin heavy variable 3 ADR*02
DRAM1	Conn	DNA damage regulated autophagy modulator 1
ESM1	Conn	Endothelial cell-specific molecule 1
TFPI	Conn	Tissue factor pathway inhibitor
MMP1	Conn	Matrix metalloproteinase 1
PXDN	Conn	Peroxidasin
KLK5	Epi	Kallikrein related peptidase 5
KRT1	Epi	Keratin 1
KRT10	Epi	Keratin 10
KRT75	Epi	Keratin 75
LCE3C	Epi	Late cornified envelope 3C
LIPM	Epi	Lipase Family Member M
ALOX12B	Epi	Arachidonate 12-lipoxygenase, 12R type
KRT2B	Epi	Keratin, type II cytoskeletal 2 oral-like
NID1	Epi	Nidogen 1
ODAM	Epi	Odontogenic, ameloblast associated
PRR9	Epi	Proline rich 9
SPINK9	Epi	Serine peptidase Inhibitor, kazal type 9
CRNN	Epi	Cornulin
DSC1	Epi	Desmocolin 1
SIRPB1	Imm	Signal regulatory proten beta 1
SLAMF6	Imm	SLAM family member 6
WFDC12	Imm	WAP four-disulfide core domain 12
CSF3R	Inflam	Colony stimulating factor 3 receptor (granulocyte)
CXCL8	Inflam	Interleukin 8
CXCL6	Inflam	Chemokine (C-X-C motif) ligand 6
HPGD	Inflam	15-Hydroxyprostaglandin dehydrogenase
PTGS2	Inflam	Prostaglandin-endoperoxide synthase 2
AKR1C3	Metab	Aldo–Keto Reductase Family 1 Member C3
EGR1	Regul	Early growth response 1
FOS	Regul	Fos proto-oncogene, AP-1 transcription factor subunit
RNU6-1	Regul	U6 spliceosome

The Adap genes are the human ID designation related to antibody nucleotide sequences of the macaque probes. Numerous of the matching macaque sequence Ig gene IDs are delineated in Thulliere et al.^75^.

Fxn denotes functional categorization of genes: *Adap* adaptive immune response, *Conn* connective tissue/bone, *Epi* epithelium, *Imm* immune response, *Inflam* inflammation, *Metab* metabolic, *Regul* regulatory, *Auto* autophagy/apoptosis, *Sig* cellular signaling, *UA* unassigned to any of these functions.

### Discrimination of gingival tissues in health and disease

**Figure 5:**
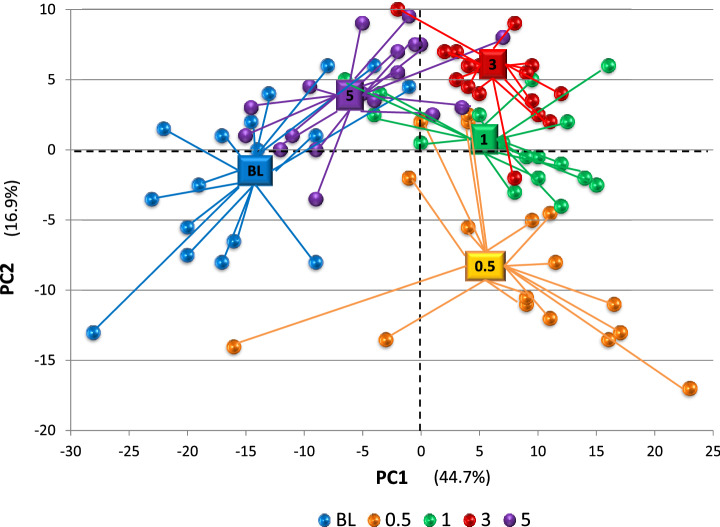
Principal components analysis of the 89 gingival tissue samples using the profile of discriminatory genes identified as disease phase-related. Each point denotes the profile of gene expression for an individual gingival tissue sample collected at baseline (healthy), 0.5 months (initiation), 1 month (early progression), 3 months (late progression), and 5 months (resolution).

We then evaluated the capacity of these subsets of differentially expressed genes to discriminate the various stages of health and disease in the gingival samples. A principal components analysis (Fig. 5) summarizes the results. These Principal Components accounted for 62% of the variation in the samples derived from the various time points. As noted the baseline healthy samples and disease initiation (0.5 months) samples demonstrated the greatest discrimination. Also, the distribution of resolution samples suggests a subset that overlapped with healthy patterns, and a second subset appeared more similar to the expression profiles for late progression (3 months). Finally, the early progression samples showed some separation from the other disease points, although the individual variation and overlap with both initiation and/or late progression, supported the limited number of unique gene patterns for the 1 month time point.

### Classification of phases of periodontitis lesions using gene expression profiles

**Figure 6:**
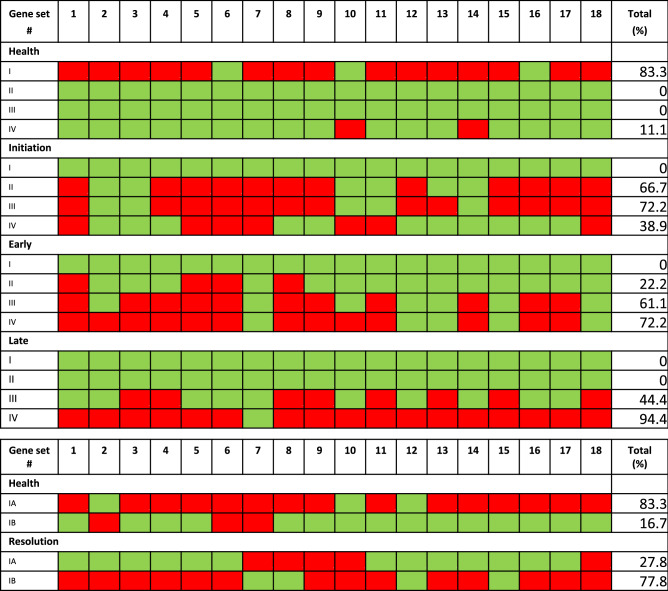
Map of response profiles for gene clusters for the 18 samples obtained at each of the timepoints. Red denotes sample demonstrated: Cluster I—> 13 genes; Cluster II—> 9 genes; Cluster III—> 7 genes; Cluster IV—> 7 genes with signal greater or less than the threshold cutoff normalized signal (T_c_, Table 3). Green denotes sample exhibited fewer genes above or below the T_c_. Similar depiction of the distribution of responses to Cluster I genes subgrouped into IA and IB. Red denotes IA—> 2 genes and IB—> 3 genes above/below the T_c_ (see Table 3). Total % denotes proportion of the samples that were positive in the gene expression cluster.

Finally, using a set of 67 genes, based upon differential expression at one or more of the timepoints, we created a flow chart to determine expression levels of each of these genes related to discriminating the phase of disease (Supplementary Fig. S1). From this measure, we determined threshold values for the ratios that were distinctive for the healthy and disease phase samples. We then determined for each sample, the number of expression values for individual gingival samples that fell above or below the threshold. This resulted in 4 different gene clusters (I-IV) represented by 20, 12, 16 and 18 genes respectively (Table 4). Additionally, subgrouping the Cluster I genes into IA and IB with a different threshold (Table 4) was effective in differentiating the baseline healthy samples to those tissues with clinically resolved lesions. Figure 6 provides a summary of the 18 specimens collected at each of the 5 time points. As noted Clusters I-IV identified health from disease with 15/18 health and only 3/18 were misclassified. Cluster II-IV gene response profiles accurately categorized 15/18 samples at disease initiation (2 weeks) and 17/18 samples at the time of late disease progression (3 months). Of interest was using these 3 clusters of responses, the early progressing lesions demonstrated a very mixed pattern of responses with 2 samples classified with health, 4 samples classified with initiation, 9 samples classified with early progression, and 3 classified with late progression specimens. The results suggest that the early progression of the lesion appears to vary biologically across the individual animals in response to the ligature challenge. Figure 6 also demonstrates the ability of the response profile of genes in Cluster IA and IB to classify what appears to be a different biology of the baseline healthy samples to those from teeth that had lesions that had clinically resolved. Using the adjusted thresholds, 13/18 healthy and 11/18 resolution samples were accurately classified.

**Table 4:** Gene clusters used to categorize the gingival samples.

Gene cluster							
I	T_c_	II	T_c_	III	T_c_	IV	T_c_
TNFRSF19	> 150	ADAM12	> 1200	DYSF	> 400	CDSN	> 1300
ANAX3	> 200	PLAT	> 150	KYNU	> 80	CEACAM8	> 600
SERPINE2	> 400	MIR223	> 400	SLC2A14	> 500	LCE3C	> 200
CD177	> 425	NOX4	> 500	CXCR1	< 500	NID2	> 1500
CSF3R	< 350	BLK	> 150	SERPINB5	> 500	THBS1	> 100
ALOX12B	> 300	KRT1	> 75	KRT24	> 250	COL15A1	> 100
KRT2B	> 100	MUC4	> 800	MZB1	> 175	COL4A1	> 500
NID1	> 50	TLR4	> 350	EGR1	> 65	SERPINE1	> 400
MMP1	> 300	HAS2	> 225	CD36	> 200	TGM2	> 1100
PXDN	< 125	TEK	> 200	PTGS2	> 400	HGF	< 350
CRNN	> 300	CTSL	> 350	LST1	> 400	TREM1	< 750
DSC1	> 300	RUBCNL	> 150	COL4A2	> 300	SELL	> 20
DRAM1	> 60			GREM1	> 450	CLDN10	> 200
ESM1	< 175			FPR3	> 350	SFRP4	> 325
TFPI	< 1000			MZB1	> 1000	SNORD116	> 150
SIRPB1	< 1300			CYP4F3	> 225	TENT5C	> 1700
HPGD	> 350					BANK1	> 150
CXCL8	< 200					BCAT1	> 75
CXCL6	> 100						
SLAMF6	> 600						

IA	T_**c**_	IB	T_**c**_				
CSF3R	< 350	CRNN	< 300				
ALOX12B	> 350	DSC1	< 300				
KRT2B	> 125	DRAM1	< 50				
NID1	> 35	ESM1	> 200				
MMP1	> 375	TFPI	< 750				
PXDN	> 125	SIRPB1	> 1300				
		HPGD	< 350				
		CXCL8	> 250				
		CXCL6	< 100				
		SLAMF6	< 700				

The threshold cutoff ratio determined as T_c_ for the normalized signal.

## Discussion

Critical features of the underlying biological processes that occur during the transition from periodontal health to disease remain elusive. It is clear that accompanying the clinical changes that hallmark this site specific mucosal disease, numerous biomarkers of inflammation and altered innate and adaptive immune response parameters can be detected in the subgingival sulcus via gingival crevicular fluid^32–35^, and even in whole saliva^36–38^ reflecting these local changes. While an array of reports using rodent models of disease have attempted to document the various host response components that contribute to the clinical changes, these models are limited by a lack of any similarity to the oral microbiome in human disease. Moreover, they generally use a biased host response assessment focusing on individual or a finite array of factors of interest. Papapanou and colleagues^39–41^ have reported a range of studies of human gingival tissue transcriptomes comparing chronic to aggressive disease, and attempting to identify crucial pathways and transcription factors that associate with existing disease. An observation from these data was the extensive heterogeneity in expression of genes in diseased tissues generally attributed to individual variation in host response genetics and the individual’s oral microbiome. However, this report describes an additional alternative for the variation in the human transcriptome profiles, that is, the human samples are obtained at a point-in-time and classified by clinical measures that could have occurred weeks/months previously, could reflect ongoing progression, or could be a disease stable site only presenting a history of a disease process. Thus, we employed a monkey model of ligature-induced disease by which we can directly identify initiation, progression, and resolution of periodontal lesions that would be difficult to accomplish in the human disease model.

This experimental model enabled us to identify transcriptomes in the gingival tissues derived from different phases of the disease process. The goal of detailing the transcriptome would be to document arrays of genes with elevated or depressed expression that discriminated healthy tissues, as well as initiation and progression of disease, and finally were uniquely expressed with disease resolution. As was expected there was a large number of genes that changed in transition from health to disease, including 900–1400 that were significantly different and either increased or decreased with disease. Additionally, there was a large number of genes that were significantly different between healthy and resolved tissues, albeit a rather limited number with substantial fold-differences.

A global transcriptomic analysis revealed a panel of genes that were specifically elevated in healthy tissues, as well as portfolios of genes that showed unique patterns at 0.5 months (initiation), 1 month (early progression), and 3 months (late progression) compared to the other time points. We also noted a panel of genes with levels elevated in resolved lesions compared to baseline or disease phases. In order to drill down more deeply within the transcriptome to identify the genes of interest, we increased the stringency for inclusion in developing these phase specific panels. This approach provided a subset of about 200 + genes that showed differential expression at the phases of disease and healthy tissues. Interestingly, at this increased level of stringency we found 53 genes in resolved tissues that differed from baseline with 59% showing decreased expression. A second step was to explore features of the panels that discriminated disease initiation from other disease phases or resolution. The results demonstrated few genes that differed uniquely between initiation and early progression. Thus, while the clinical parameters increased significantly from 2 weeks (0.5 months) to 1 month, it appears that changes in gene expression occur rapidly and are maintained during this early progression phase of disease. In contrast, we identified 104 genes that identified tissues at late disease progression versus levels in tissue samples during disease initiation with 58% of the expression levels greater in late progression. This suggested the likelihood of some specific gene profiles whose function may contribute to the continued progression of the disease lesions. Finally, a number of genes (n = 55) were decreased in resolved tissues compared to expression during any phase of disease. Most of these represented gene expression levels that were returning back towards baseline levels consistent with the improved clinical parameters.

These findings documented the existence of genes that demonstrated significant differences across the biologic phases of the disease process and enabled the creation of profiles of “phase-specific” genes. The genes elevated in health, but never in diseased tissues were over-represented for biologic pathways of epithelial cell functions, metabolism, and inflammation (Table 2). As these gene expression variations represent early disease changes in this human-like disease model, potentially a subset of these gene products could be targeted as early biomarkers of disease in humans, potentially enabling earlier identification and intervention to minimize tissue damage.

The data also explored the determination if a pattern of genes was also relatively specific for what would be considered “early progression” (1 month) in this model related to clear increases in the trajectory of clinical parameters of BOP and PPD. Interestingly, a rather broad variation in expression of the various clusters of genes was noted during early progression. In the nonhuman primate model, extensive studies of clinical measures have demonstrated a general expression of periodontal disease within 1 month post-ligation in nearly all animals although the rate differs for individual animals^42–44^. Also, a portion of the animals demonstrate clinical measures that show minimal increases between 1 and 3 months (early responders), while a subset of the animals clearly demonstrate a continued progression of disease, reaching maximum PPD at the 3 month time point, which we defined as “late progression”. As such, the gene analysis prediction grouped selected animals at early progression as either initiation, early or late progression, suggesting an extent of biologic variation in the population during the early transition to progressing destructive disease. This type of temporal variability is likely also reflected within the human population based upon clinical measures of gingivitis and progressing periodontitis, albeit little is known regarding the biological differences occurring with this disease transition. In contrast, the gene profiling analysis identified an array of genes with levels that were expressed uniquely at 3 months (late progression) and were able to discriminate this disease phase. These included immune pathways and, of particular note was the large array of gene expression changes related to formation and recombination events for antibody molecules. Moreover, some genes that were increased at 1 month continued to increase to an even greater extent at 3 months, while other members of this late progressing panel were elevated only at this phase of disease. These patterns enabled the identification of a set of 67 genes that could be assembled into 4 clusters and used to classify health and disease phases. Creating thresholds for individual gene expression, the 4 clusters effectively classified the specimens from healthy sites with > 80% accuracy and delineated the initiation and late progression samples at 72% and 94% accuracy, respectively.

Finally, the data provided insight into fundamental biologic differences between gingival tissues of periodontal health and clinically normal gingiva at sites where the lesion has resolved. In this regard, there is actually quite limited data regarding gene expression profiles in human tissues following non-surgical therapy. Beikler et al.^45^ examined a limited subset of gene encompassing inflammatory responses, as well as epithelial, connective, and endothelial tissues celluar responses. As such, our model in which we mitigate local inciting factors similar to scaling and root planing in humans provides some insights into addressing differences in healthy tissues compared to tissues at sites of resolved lesions. As might be expected, there was a limited number of genes with expression differences between healthy and resolved gingival tissues representing epithelial biology and inflammatory responses. However, using additional threshold levels for expression of Cluster I genes 75–80% of the healthy versus resolved specimens were categorized accurately. Thus, the gingival tissues do not appear to return to complete biologic health within 60 days of clinical disease resolution. This might suggest that previously diseased sites that have been treated and resolved may appear clinically normal; however, they could remain biologically programmed for a heightened risk of disease related to a subsequent noxious challenge. Similarly a recent clinical study reported that even in well-maintained patients the oral microbiome seems more pathogenic than in healthy control sites without previous history of periodontitis^46^. This finding also supports a new concept related to the recent classification scheme for periodontitis and reinforces the concept that a patient with periodontal disease experience will be always a patient with enhanced periodontal disease risk even after reaching clinically healthy conditions.

While this report focused on characterizing the gingival transcriptome in this model of health, disease, and resolution, it cannot be ignored that there also occur parallel changes in the oral microbiome with increases in pathobionts and a resulting dysbiosis^47^ that reflects and/or drives the tissue destructive features of periodontitis. Importantly, there exists a reasonable linkage between reported microbiome changes and the features of gene expression changes that were identified. *P. gingivalis* hallmarks these changes by impacting apoptosis and autophagy processes allowing it to invade, survive, and disrupt the normal lifecycle of epithelial cells^48,49^. Additionally, other reports have documented dysregulated inflammatory, protective, and cellular integrity for an array of cells induced by numerous oral bacteria^50,51^. Related to the array of genes that were altered with disease initiation and progression, a number of proposed oral pathogens also have the capacity to translocate to deeper tissues and create intraepithelial/mucosal microbial communities. This process would trigger an enhanced inflammatory cell infiltrate and resulting microvascularization as a feature of the inflammatory response in disease^52^. As such, the dysbiotic microbiome, related to an increased burden of individual pathogenic species, undermines the normal homeostatic mechanisms of the various gingival cells, with genes in the phase arrays from this study consistent with these microbial-induced changes^53^.

This model provided us the capacity to develop algorithms of gene expression that enabled phasing disease and distinguish between resolved and uninvolved healthy sites. Based upon existing data from cross-sectional human gingival transcriptomes^54–57^, we can test these gene panels with the human data to potentially enhance the homogeneity of the human specimens for disease phases and resolution and identify with more precision biomarkers that may be useful in management of the human disease. However, utility of this specific gene expression knowledge would be limited for clinical care in humans since routine sampling of gingival tissues for targeted gene expression would not be feasible. Nevertheless, many of these genes would be predicted to result in secreted translated biomolecules that would be expected to be present in the gingival crevicular fluid and could even be detected in saliva to potentially discriminate health, disease phases, and resolved lesions. Importantly, as shown in Table 5 many of these biomolecules have already been evaluated and associate with many chronic inflammatory conditions. Based upon this unbiased approach to identification of potential biomarkers, panels of a finite number of gene products could be evaluated in humans to enable a better understanding of molecular mechanisms and development of targeted therapies with more precision.

**Table 5:** Targeted biomarkers for periodontal lesion phases reported as diagnostic biomarkers for various inflammatory and non-inflammatory diseases.

Cluster	Protein	Fluid	Biologic linkage
I	ALOX12B	Serum/plasma	Diabetes
I	CRNN	Serum/plasma	Epithelial-induced stress protein
I	CSF3R	Serum/plasma	Cancer related gene
I	CXCL6	Serum/plasma/urine/saliva	Antibacterial/neutrophil
I	CXCL8 (IL-8)	Serum/plasma/saliva	Pancreatic cancer; breast cancer
I	ESM1	Serum/saliva	(Endocan) CVD, PCOS
I	HPGD	Serum/plasma/saliva	Cancer
I	MMP1	Serum/plasma/saliva	CVD; arthritis
I	NID1	Plasma/saliva	Cancer
I	PXDN	Serum/plasma/saliva	Fibrosis
I	TFPI	Serum/plasma	Coagulation
I	TNFRSF19	Serum/plasma	Cancer
I	SERPINE2	Serum	Cancer
II	TLR4	Serum/plasma/saliva	Arthritis, autoimmunity
II	ADAM12	Serum/plasma	Fibrosis; lung disease
II	KRT1	Serum/plasma	Cancer
II	MIR223	Serum/plasma/saliva	Cancer; CVD
II	MUC4	Serum/cyst fluid/saliva	Cancer
II	NOX4	Serum/plasma	Inflammation
II	PLAT	Serum/plasma/saliva	CVD
II	TEK	Serum/plasma	Cancer, autoimmune
II	HAS2	Serum/plasma/saliva	Inflammation; arthritis
III	CD36	Serum/plasma	Foam cell formation (CVD)
III	KYNU	Serum/plasma	Psoriasis
III	GREM1	Serum	Inflammation
III	FPR3	Serum	COPD
IV	SERPINE1	Serum/plasma/saliva	CVD (PAI-1)
IV	CEACAM8	Serum/plasma/saliva	Arthritis
IV	HGF	Serum/plasma/saliva	Liver disease; cancer
IV	NID2	Serum/plasma/saliva	Cancer
IV	SELL	Plasma	Alzheimer’s; schizophrenia
IV	SFRP4	Serum/plasma	Diabetes
IV	THBS1	Serum/saliva	Obesity; pregnancy
IV	TREM1	Serum/saliva	Inflammatory bowel disease

## Methods

### Animal model of periodontitis

Rhesus monkeys (*Macaca mulatta*) (n = 18; 10 females and 8 males) aged 12–23 years housed at the Caribbean Primate Research Center at Sabana Seca, Puerto Rico^58–60^. The nonhuman primates were typically fed a 20% protein, 5% fat, and 10% fiber commercial monkey diet (diet 8773, Teklad NIB primate diet modified: Harlan Teklad, Madison, WI). The diet was supplemented with fruits and vegetables, and water was provided ad libitum in an enclosed corral setting.

All experimental protocols were approved by the Institutional Animal Care and Use Committees (IACUC) of the University of Puerto Rico and University of Kentucky. The methods were carried out in accordance with all relevant regulations for the use of nonhuman primates following ARRIVE guidelines. Anesthetized animals were examined by a single investigator using a Maryland probe on the facial aspect of the teeth, 2 proximal sites per tooth (mesio- and disto-buccal), excluding the canines and 3rd molars. The clinical examination included probing pocket depth (PD), and bleeding on probing (BOP; 0–5 scale)^16^. Periodontal health was defined by mean Pocket Depth (PD) ≤ 3.0 mm and mean Bleeding on Probing (BOP) ≤ 1 (0–5 scale) in a full mouth examination excluding 3rd molars and canines^61^. Determination of periodontal disease at the sampled site was documented by assessment of the presence of BOP and probing pocket depth of > 4 mm as we have described previously.

Ligature-induced periodontitis was induced in each of the animals at 1st premolar and 1st and 2nd molars in all 4 quadrants following a baseline sampling of gingival tissue from a healthy site. Further, clinical evaluation for ligated sites was obtained and a buccal gingival papilla from each animal was taken using a standard gingivectomy technique at 2 weeks (initiation of disease), and 1 month and 3 months (progression of disease). Then, ligatures were removed after sampling at 3 months and samples taken 2 months later (resolution)^28,62,63^. Since the removal of the ligature eliminates the local noxious mechanical challenge and decreases the microbial burden accumulating at the tooth, this process is similar to nonsurgical periodontal therapy in humans. Previously published histological studies have documented the significant increase in inflammatory cell infiltrate in the ligated tissues consistent with the clinical features of inflammation and increased probing pocket depth^64,65^.

### Gingival tissue sample collection and mRNA analysis

Gingival tissue samples of healthy of disease sites were surgically collected as we have described previously providing buccal gingival samples from either healthy or periodontitis-affected tissue from the premolar/molar maxillary region of each animal using a standard gingivectomy technique^58,66,67^. Samples were maintained frozen at − 80 °C in RNAlater solution until RNA preparation for microarray analysis. Total RNA was isolated from tissues using TRizol reagent (Invitrogen, Carlsbad, CA, USA). After cleaning with Qiagen RNeasy mini kit (Qiagen, Valencia, CA, USA), all microarray RNA expression analyses were done at the University of Kentucky Microarray facility. Tissue RNA samples were submitted to the UK Microarray Core Facility and RNA quality was assessed with an Agilent 2100 Bioanalyzer (Agilent Technologies, Santa Clara, CA, USA). Reverse transcription of equal amounts of RNA from each sample was performed, followed by hybridization to the GeneChip Rhesus Gene 1.0 ST Array (Affymetrix, Santa Clara, CA, USA) similar to methods we have described previously^59,67,68^. We have also previously published multiple reports examining an array of biologic pathways and included validation of numerous genes between the microarray and qPCR results^61,64,65,69–71^. A summary of these results is displayed in Supplemental Fig. S2 that demonstrates, as we have noted previously, a significant relationship between the microarray and qPCR analyses regarding directional changes, albeit the qPCR tended to provide a somewhat broader range in the differential expression changes.

### Data analysis

The expression intensities for genes across the 18 samples were estimated using the Robust Multi-array Average (RMA) algorithm with probe-level quintile normalization, as implemented in the Partek Genomics Suite software version 6.6 (Partek, St. Louis, MO). The different groups were initially compared using one-way ANOVA. For genes that had significant mean differences, two sample t-tests were used to investigate differences. The data has been uploaded into the ArrayExpress data base (www.ebi.ac.uk) under accession number: E-MTAB-1977. A number of Affymetrix probes with unique expression profiles in the samples had not been annotated. For these, we used the Ensembl ID (EMBL-EBI) to extract out the nucleotide base sequence for the probe from the Affymetrix Exon/Gene (http://www.affymetrix.com/analysis/index.affx#1_2) website. This sequence was then searched in Blast (https://blast.ncbi.nlm.nih.gov/Blast.cgi) and the highest percent identity for *M. mulatta* selected.

Normalized expression profiles of genes that were differentially expressed at least across one of the time points Baseline, 2 weeks, 1 Month, 3 Months, 5 Months with the remaining time points as background were used as input to Principal component analysis (PCA)^72–74^. The first and second dominant eigen values explained ~ 62% of the variance in the given data and two-dimensional projection of the gene expression profile revealed inherent clustering of the time points.

## Supplementary Information

Supplementary Information 1.

Supplementary Information 2.
