# Supplementary material for: Transcriptomic phases of periodontitis lesions using the nonhuman primate model
Source: Sci Rep. 2021 Apr 29;11:9282. doi: 10.1038/s41598-021-88803-6 (PMC8085193; doi:10.1038/s41598-021-88803-6)
Supplement: Supplementary file 1 — Supplementary Information 1. [file 41598_2021_88803_MOESM1_ESM.docx]

**Supplemental Figure 1**: Flow of specific gene selection for discriminating health, phases of disease, and resolution.

**Supplemental Figure 2:** Depiction of validation of microarray data for a set of 24 genes using qPCR. The genes included in the comparison were: ATG4A, IF130, IL1A, IL1B, IL6, IL8, CCL19, CXCL13, CXCR4, CD14, CLEC7A, CTSS, DAI/ZBP1, ENOL1, EPAS1, FOS, LDHA, MMP9, NLRP14, NOD2, PGAM1, SAA1, SPP1, and TLR2. Characteristics of the linear regression and significance value are included.
