# Supplementary material for: Transcriptomic phases of periodontitis lesions using the nonhuman primate model
Source: Sci Rep. 2021 Apr 29;11:9282. doi: 10.1038/s41598-021-88803-6 (PMC8085193; doi:10.1038/s41598-021-88803-6)
Supplement: Supplementary file 2 — Supplementary Information 2. [file 41598_2021_88803_MOESM2_ESM.pptx]

## Slide 1
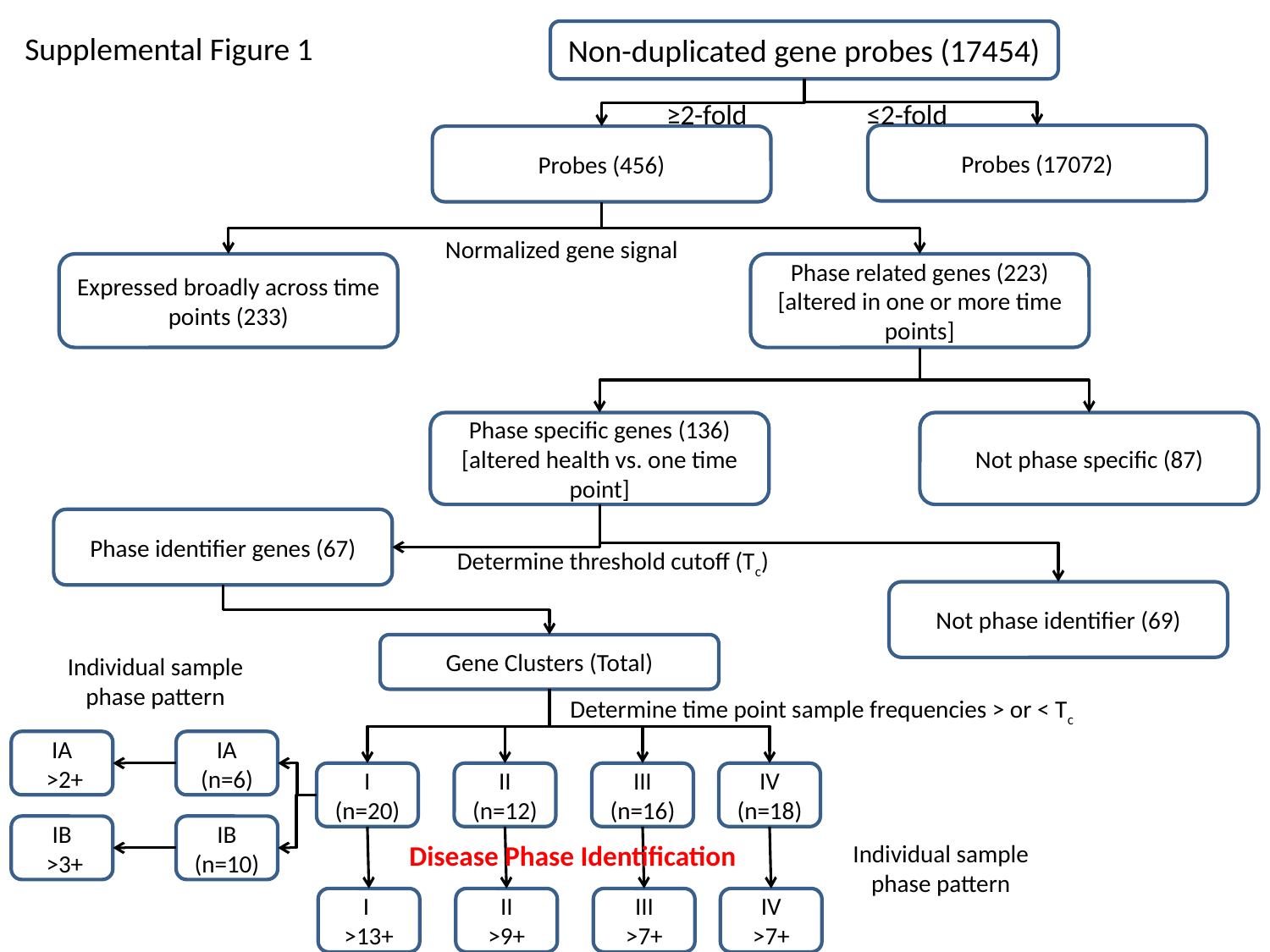

Non-duplicated gene probes (17454)
Supplemental Figure 1
≥2-fold
≤2-fold
Probes (17072)
Probes (456)
Normalized gene signal
Expressed broadly across time points (233)
Phase related genes (223)
[altered in one or more time points]
Phase specific genes (136)
[altered health vs. one time point]
Not phase specific (87)
Phase identifier genes (67)
Determine threshold cutoff (Tc)
Not phase identifier (69)
Gene Clusters (Total)
Individual sample
phase pattern
Determine time point sample frequencies > or < Tc
IA
 >2+
IA (n=6)
I (n=20)
II
(n=12)
III
(n=16)
IV
(n=18)
IB
 >3+
IB (n=10)
Disease Phase Identification
Individual sample
phase pattern
I
>13+
II
>9+
III
>7+
IV
>7+

## Slide 2
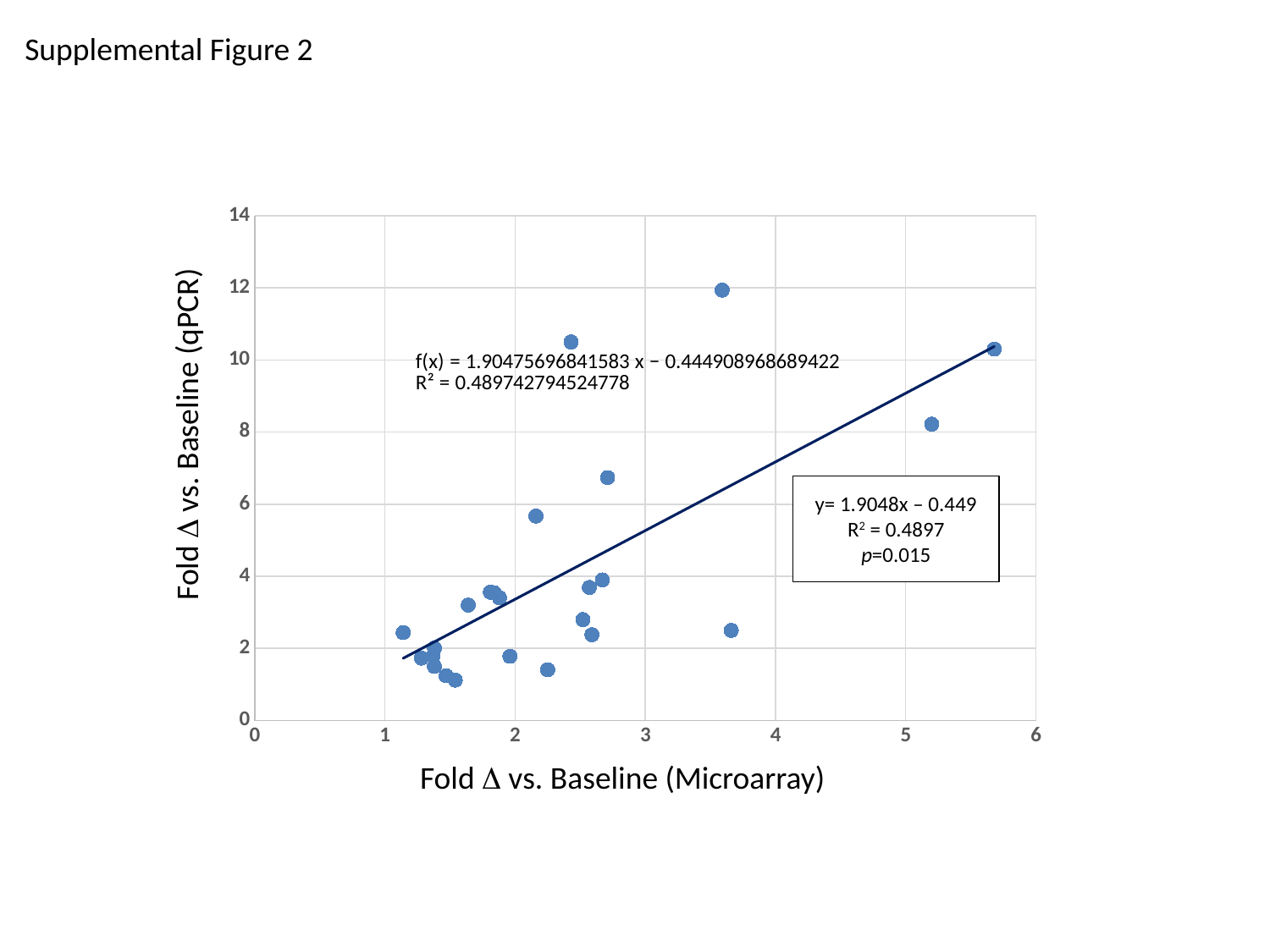

Supplemental Figure 2
### Chart
| Category | Y-Values |
|---|---|Fold D vs. Baseline (qPCR)
y= 1.9048x – 0.449
R2 = 0.4897
p=0.015
Fold D vs. Baseline (Microarray)
